# Validation of thigh-based accelerometer estimates of postural allocation in 5–12 year-olds

**DOI:** 10.1016/j.jsams.2016.08.008

**Published:** 2017-03

**Authors:** Christiana M.T. van Loo, Anthony D. Okely, Marijka J. Batterham, Trina Hinkley, Ulf Ekelund, Søren Brage, John J. Reilly, Rachel A. Jones, Xanne Janssen, Dylan P. Cliff

**Affiliations:** a Early Start Research Institute and Illawarra Health and Medical Research Institute, Faculty of Social Sciences, University of Wollongong, Australia; b School of Mathematics and Applied Statistics, University of Wollongong, Australia; c Deakin University, Geelong, Australia, Institute for Physical Activity and Nutrition (IPAN), School of Exercise and Nutrition Sciences; d Norwegian School of Sports Sciences, Norway; e MRC Epidemiology Unit, University of Cambridge, United Kingdom; f University of Strathclyde, School of Psychological Sciences and Health, Scotland

**Keywords:** Sedentary behaviour, Physical activity, Child, Accelerometry, activPAL, Breaks

## Abstract

**Objectives:**

To validate activPAL3™ (AP3) for classifying postural allocation, estimating time spent in postures and examining the number of breaks in sedentary behaviour (SB) in 5–12 year-olds.

**Design:**

Laboratory-based validation study.

**Methods:**

Fifty-seven children completed 15 sedentary, light- and moderate-to-vigorous intensity activities. Direct observation (DO) was used as the criterion measure. The accuracy of AP3 was examined using a confusion matrix, equivalence testing, Bland–Altman procedures and a paired *t*-test for 5–8y and 9–12y.

**Results:**

Sensitivity of AP3 was 86.8%, 82.5% and 85.3% for sitting/lying, standing, and stepping, respectively, in 5–8y and 95.3%, 81.5% and 85.1%, respectively, in 9–12y. Time estimates of AP3 were equivalent to DO for sitting/lying in 9–12y and stepping in all ages, but not for sitting/lying in 5–12y and standing in all ages. Underestimation of sitting/lying time was smaller in 9–12y (1.4%, limits of agreement [LoA]: −13.8 to 11.1%) compared to 5–8y (12.6%, LoA: −39.8 to 14.7%). Underestimation for stepping time was small (5–8y: 6.5%, LoA: −18.3 to 5.3%; 9–12y: 7.6%, LoA: −16.8 to 1.6%). Considerable overestimation was found for standing (5–8y: 36.8%, LoA: −16.3 to 89.8%; 9–12y: 19.3%, LoA: −1.6 to 36.9%). SB breaks were significantly overestimated (5–8y: 53.2%, 9–12y: 28.3%, *p* < 0.001).

**Conclusions:**

AP3 showed acceptable accuracy for classifying postures, however estimates of time spent standing were consistently overestimated and individual error was considerable. Estimates of sitting/lying were more accurate for 9–12y. Stepping time was accurately estimated for all ages. SB breaks were significantly overestimated, although the absolute difference was larger in 5–8y. Surveillance applications of AP3 would be acceptable, however, individual level applications might be less accurate.

## 1 Introduction

High levels of sedentary behaviours (SB) and prolonged bouts of SB are negatively associated with health outcomes in adults,^1^, ^2^ independent of the amount of time engaged in moderate-to-vigorous intensity physical activity (MVPA).^3^ Frequent interruptions in sedentary time could reduce this risk.^4^, ^5^ Although some studies among children and adolescents^6^, ^7^, ^8^ suggest that the total volume or pattern of SB is associated with adverse health outcomes, overall, the evidence among young age groups is inconsistent.^9^, ^10^, ^11^ The accurate measurement of SB in observational and experimental research in children is essential to better understand the potential influence of SB on health outcomes.

Assessing subtle differences between SB and light-intensity physical activity (LPA) using traditional hip-mounted accelerometers and cut-point methodologies seems to be difficult, because these methods categorise SB based on the lack of movement,^12^ and some LPAs such as standing tend to be misclassified as SB.^13^, ^14^ Activity monitors or data reduction approaches that are sensitive to changes in posture offer potential for improved measurement of SB and LPA. An example is the activPAL3™ (AP3; PAL Technology Ltd., Glasgow, Scotland), an activity monitor worn on the thigh that uses triaxial acceleration data (20 Hz) to assess the position and movement of the limb. The AP3 software uses proprietary algorithms to classify periods spent sitting/lying, standing or stepping. Before being used in observational and experimental studies in children, it is important to determine if the device accurately detects postures and precisely estimates time spent sedentary and non-sedentary. Furthermore, it is important to evaluate the device’s accuracy to detect breaks in SB in order to understand their influence on health outcomes.

The uni-axial activPAL™ (AP1) has been validated in young children (3–6y),^15^, ^16^, ^17^ but to our knowledge only one study has evaluated AP1 in school-aged children.^18^ Aminian et al.^18^ included 25 participants aged 9–10y who performed 4 sedentary and 7 ambulatory activities, plus a selection of 3 activity patterns including sit-to-stand and stand-to-sit transitions to simulate real-world conditions. High correlations were found between direct observation (DO) and time spent in different postures and transitions between postures, as estimated by AP1. However, correlational approaches can only determine the relative strength of the relationship between measurement outcomes and do not provide information about potential systematic differences or the agreement between estimates.^19^, ^20^ Data on the measurement agreement or potential systematic bias of the monitor was only reported in 4–6y.^16^ No studies have investigated whether potential measurement errors of the monitor lie within a clinically acceptable range. This study aimed to examine the classification accuracy and validity of AP3 for estimating sitting/lying, standing and stepping time and the number of SB breaks in 5–12 year-old children.

## 2 Methods

Fifty-seven children (5–12y) who were without physical or health conditions that would affect participation in physical activity were recruited. The study was approved by the University of Wollongong Health and Medical Human Research Ethics Committee. Parental written consent and participant verbal assent were obtained prior to participation.

Participants were required to visit the laboratory on two occasions. Anthropometric measures were completed using standardised procedures after which BMI (kg/m^2^) and weight status were calculated.^21^ Children completed a protocol of 15 semi-structured activities (Supplementary Table 1) from sedentary (e.g. TV viewing, writing/colouring), light (e.g. slow walk, dancing), and moderate-to-vigorous (e.g. soccer, running) intensity. Activities were equally divided over 2 visits and completed in a structured order of increasing intensity for 5 min, except for lying down (10 min).

The single unit accelerometer AP3 (53 × 35 × 7 mm, 15.0 g) was placed mid-anteriorly on the right thigh and initialised with minimum sitting or upright period of 1 s. Event records created by the AP3 software were used to classify periods spent sitting/lying, standing or stepping and transitions from sit/lie to upright (breaks in SB).

DO was used as the criterion measure. Children were recorded on video completing the activities as well as during transitions between activities. A single observer coded all videos using Vitessa 0.1 (University of Leuven, Belgium) which generated a time stamp every time a change in posture was coded. Subsequently, a second-by-second classification system was generated using customised software, in order to synchronise DO data with AP3’s 1s epochs. Every second following the time stamp inserted by the observer was classified the same as the posture occurring at the time stamp itself until the next time stamp was created, indicating that the child’s posture had changed. Postures were coded as sitting/lying (gluteus muscles resting on ground, feet, legs or any other surface, or lying in prone position), standing (both feet touching the ground), “other standing” (e.g. squatting, standing on one foot, kneeling on one or two knees), stepping (moving one leg in front of the other, including stepping with a flight phase), “other active” (e.g. jumping, sliding/side gallop) and “off screen” for DO. Seconds coded as “other standing” were recoded as standing, because these postures required the engagement of large postural muscles and did not involve the gluteus muscles resting on any surface. Seconds coded as “other active” were recoded as stepping. In the event of two postures occurring within the same second in either DO or AP3 data, this second was duplicated at the corresponding time point for the AP3 or DO output, in order to evaluate classification accuracy. This method was in line with previous validation studies.^15^, ^16^ For estimated time spent in postures, codes of duplicated seconds for either DO (0.02% of total DO data) or AP3 (0.04% of total AP3 data) were assigned 0.5 sec to avoid artificially inflating the total time observed. The synchronised DO and AP3 epochs were excluded when DO was coded as “off screen”, which occasionally occurred when moving between different locations during transitions. Videos of 5 randomly selected participants were analysed twice by the same observer and once by a criterion observer to test inter- and intra-observer reliability. Inter- and intra-observer reliability were examined using Cohen’s Kappa and single measure intra-class correlation coefficients (ICC) from two-way mixed effect models (fixed-effects = observer; random effects = participants), using the consistency definition. Cohen’s Kappa coefficient for inter-observer reliability was 0.941. Inter-observer ICC was 0.974 (0.974–0.974) and intra-observer ICC was 0.963 (0.962–0.963).

Prior to analyses, participants were divided into two age groups (5–8y and 9–12y) because younger and older children potentially engage in and move between sitting, standing and non-standard postures differently.^16^, ^22^ Normality of the data was confirmed and analyses were performed for each group. The accuracy of AP3 for classifying sitting/lying, standing and stepping was established using sensitivity (true positive rate) and specificity (true negative rate), and summarised using a confusion matrix.^23^ The equivalence of time estimates between AP3 and DO for each posture was examined at the group level using the 95% equivalence test. The methods are equivalent if the 90% confidence interval (CI) of time estimated by AP3 entirely falls within the predefined equivalence region of ±10% of the average time coded by DO.^24^, ^25^ Measurement agreement and systematic bias for estimated time spent in postures were evaluated at the individual level using Bland–Altman procedures.^20^ Pearson correlations were used to evaluate the ability of AP3 to estimate the relative number of SB breaks compared to DO. The difference between the absolute number of SB breaks was tested using a paired sample *t*-test. Analyses were performed using the statistical computing language R v.3.1.2 and SPSS v.19.0.

## 3 Results

Descriptive characteristics of participants are presented in Supplementary Table 2. All participants completed the protocol and had valid AP3 data. Videos from one of the visits were unavailable for 3 children (age 5, 9 and 10y). Out of the remaining 267,952 1s epochs of DO from 5 to 8y and 345,226 epochs from 9 to 12y, 27,493 epochs and 25,042 epochs were coded as “off screen” and excluded from analyses, respectively, leaving 240,459 (89.7%) valid epochs for 5–8y and 320,184 (92.7%) for 9–12y. Mean DO time for 5–8y was 167.0 ± 22.4 min, of which 77.8 ± 12.0 min was classified as sitting/lying, 26.9 ± 8.6 min as standing and 62.2 ± 9.3 min as stepping. Mean DO time for 9–12y was 161.8 ± 26.1 min, of which 73.0 ± 14.3 min, 26.3 ± 8.7 min and 62.5 ± 10.5 min were classified as sitting/lying, standing and stepping, respectively.

The sensitivity and misclassifications for AP3 are presented in Table 1. Sensitivity of 86.8%, 82.5% and 85.3% in 5–8y was acceptable for sitting/lying, standing and stepping, respectively. In 9–12y, sensitivity of 95.3% was excellent for sitting/lying and sensitivity of 81.5% and 85.1% was acceptable for standing and stepping, respectively. Specificity was 98.0%, 87.7% and 95.1%, for sitting/lying, standing and stepping in 5–8y, respectively, and 97.8%, 92.0% and 94.7% in 9–12y, respectively. Sitting/lying was misclassified as standing for 11.8% of the time in 5–8y, whereas this was only 3.6% in 9–12y. 14.8% and 16.8% of standing was misclassified as stepping for 5–8y and 9–12y, respectively. Furthermore, 13.0% and 13.1% of stepping were misclassified as standing for 5–8y and 9–12y, respectively.

**Fig. 1:**
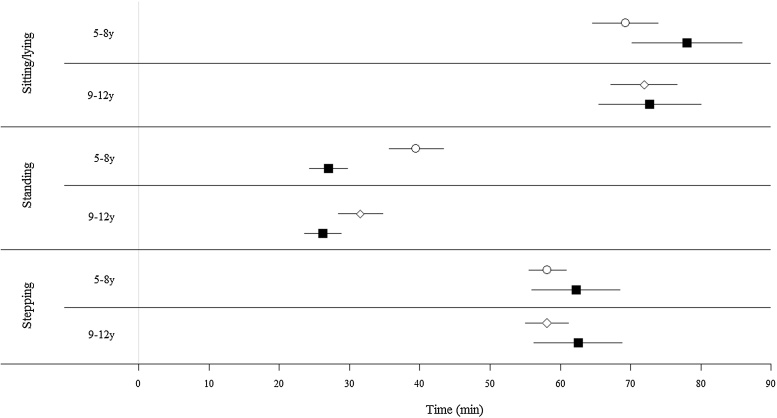
95% equivalence test for estimated time spent sitting/lying, standing and stepping. Times estimated by activPAL3™ (AP3) are equivalent to direct observation (DO) if 90% confidence intervals lie entirely within the equivalence region of direct observation. AP3: ○ = 5–8y, ♢ = 9–12y; DO: ■.

**Fig. 2:**
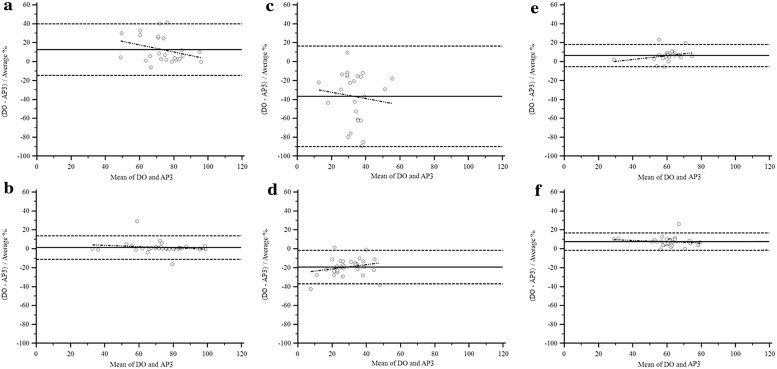
Bland–Altman plots. Bland–Altman plots with 95% limits of agreement for time spent sitting/lying (a: 5–8y, b: 9–12y), standing (c: 5–8y, d: 9–12y) and stepping (e: 5–8y, f: 9–12y). DO: direct observation, AP3: activPAL3™. Mean bias was calculated as percentages proportionally to the magnitude of the measurements using DO-AP3; a positive value indicates underestimation of time spent in the posture by AP3; a negative value indicates overestimation of time spent in the posture by AP3.

At the group level (Fig. 1), estimates of AP3 were equivalent to DO for sitting/lying time in 9–12y (*p* < 0.001) and stepping time in both age groups (5–8y, *p* = 0.004; 9–12y, *p* = 0.001). Estimated sitting/lying time in 5–8y and standing time in both age groups were not equivalent to DO (*p* > 0.05). Bland–Altman procedures (Fig. 2) demonstrated underestimation for sitting/lying time in both age groups. The mean difference in 5–8y was 12.6% (limits of agreement [LoA]: −39.8 to 14.7%), however the difference and LoA in 9–12y were considerably smaller (1.4%, LoA: −13.8 to 11.1%). Stepping time was underestimated in both age groups (5–8y, mean difference: 6.5%, LoA: −18.3 to 5.3%; 9–12y, mean difference: 7.6%, LoA: −16.8 to 1.6%), whereas the overestimation for standing time was considerably larger (5–8y, mean difference: 36.8%, LoA: −16.3 to 89.8%; 9–12y, mean difference: 19.3%, LoA: −1.6 to 36.9%). At the individual level, LoAs were notably wider for sitting/lying and standing time in 5–8y, whereas LoA for stepping time was similar for both age groups. No systematic bias was found for the postures (*p* > 0.05). Although the correlation of the number of SB breaks detected by AP3 was significant (5–8y, Pearson’s *r* = 0.73, *p* < 0.001; 9–12y, Pearson’s *r* = 0.81, *p* < 0.001), the absolute number of breaks was overestimated for both age groups, but more so for 5–8y (AP3: 24.2 ± 8.6, DO: 15.8 ± 4.6, *p* < 0.001) than 9–12y (AP3: 15.4 ± 5.1, DO: 12.0 ± 3.4, *p* < 0.001).

## 4 Discussion

AP3 demonstrated acceptable sensitivity and specificity for classifying postures in both age groups. Time spent sitting/lying and stepping was slightly underestimated in 5–8y (∼6–13%) and 9–12y (∼2–8%), however measurement errors lay within a conventional range of ±10% of the criterion for sitting/lying time in 9–12y and for stepping time in both age groups. Standing time was overestimated in both younger (36.8%) and older (19.2%) children and was not equivalent to DO. At the individual level, wide LoA was found for sitting/lying time and very wide LoA for standing time in 5–8y. Less individual variability was found for sitting/lying time in 9–12y, however the LoA for standing in this age group was also considerably wide. The absolute number of breaks in SB was statistically overestimated by AP3, although the difference for 9–12y (28.3%) was smaller than for 5–8y (53.2%). A significant correlation was present between breaks detected by AP3 and DO in both age groups.

Aminian et al.^18^ reported a perfect correlation (*r* = 1.00) between AP1 and DO for time spent sitting/lying, standing and walking including activity patterns, and a high correlation for transition counts (*r* = 0.99). However, no information was presented on potential measurement errors and/or systematic bias. Although the accurate assessment of postural allocation in our study was in line with the high correlation between AP1 and DO in the previous study, AP3 estimated time spent standing less accurately and the individual-level error for time spent sitting/lying in 5–8y and standing in both age groups was substantial.

Compared to previous studies that tested AP1 in preschoolers, the sensitivity of AP3 for sitting/lying was similar to Janssen et al.^16^ (87.6%) in 5–8y (86.8%), and similar to Davies et al.^15^ (92.8%) in 9–12y (95.3%). However, sitting/lying in our sample was classified more accurately in both age groups compared to SB (sensitivity: 53.8%) reported by De Decker et al.^17^ Sensitivity of AP3 for standing in our sample (5–8y: 82.5%, 9–12y: 81.3%) was lower compared to Davies et al.^15^ (91.8%), but higher than Janssen et al.^16^ (75.6%). Sensitivity for stepping (5–8y: 85.3%, 9–12y: 84.6%) was higher compared to both Davies et al.^15^ (77.9%) and Janssen et al.^16^ (52.5%). Errors for estimates of time spent in postures in our sample were slightly different to those in studies of preschoolers. Overall errors for sitting/lying were small in 9–12y in our study (1.4%), as well as in Davies et al.^15^ (−4.4%) and Janssen et al.^16^ (5.9%), whereas sitting/lying time in 5–8y in our study was underestimated by 12.6%. The minimal error for stepping time in our sample was consistent with errors in preschoolers (no difference^15^ and 10.0%^16^). The monitor overestimated standing time in all studies, although the overall errors in preschoolers were smaller (7.1%^15^ and 10.0%^16^, respectively) compared to 5–8y (36.8%) and 9–12y (19.3%) in the current sample. The authors of those studies suggested that misclassifications can be related to sitting being misclassified as standing by AP1,^15^, ^16^ which could explain the relatively large individual error for sitting/lying time in 5–8y and standing time in both age groups in our study. We further investigated the videos and discovered that children for whom sitting/lying was overestimated the most were 5–8y. These participants were seated on the edge of a chair with legs outstretched during the rest periods between activities, causing AP3 to misclassify the posture as standing. This aligns with previous reports^15^, ^16^ suggesting that the non-standard postures that children sometimes engage in might influence sit/lie misclassification by the monitor.

The absolute number of SB breaks estimated by AP3 in our study was significantly overestimated by 8.4 breaks (53.2%) in 5–8y and 3.4 breaks (28.3%) in 9–12y. AP1 also overestimated the number of SB breaks among preschoolers by 43.6%^16^ and 66.7%.^22^ The authors suggested that this was related to the impact of non-standard postures on the estimates of SB breaks. Davies et al.^22^ and Janssen et al.^16^ noted that 34.0% and 63.8% of transitions, respectively, were from non-standard postures to upright postures. The number of transitions from “other standing” to upright postures in our study was 23.2% of the total number of transitions in 5–8y and 36.5% in 9–12y, which might not explain the larger overestimation of breaks in 5–8y. However, the definitions of non-standard postures in previous studies^16^, ^22^ included both non-standard sitting and non-standard standing. Because numerous non-standard postures identified in previous research^22^ appeared to be more similar to standing than sitting, in that they required the activation of large postural muscles (e.g. crouching and kneeling up), these were classified separately in our methods as “other standing”. After visual inspection of the videos, non-standard sitting postures, which were not coded separately in our study, may have contributed to the overestimation of SB breaks. For example, if the child was sitting on a chair with thigh parallel to the ground and moved to the edge of the chair with legs outstretched (non-standard-sitting), AP3 may have classified this movement as an additional break, relative to DO. As suggested by Davies et al.,^22^ the relative assessment of the number of SB breaks may be more important than the absolute number for epidemiological applications to understand the physiological and health consequences of the breaks. In agreement with previous studies in school-aged^18^ and preschoolers,^22^ our study demonstrated a significant correlation for SB breaks assessed by AP3 and DO in both age groups, indicating that AP3 is accurate when evaluating the relative number of breaks.

The strengths of this study include the relatively larger sample and the wider age-range of participants compared to previous studies.^15^, ^16^, ^18^ Furthermore, a wider range of non-ambulatory activities was included compared to the activity protocol used previously with school-aged children.^18^ Data from the entire activity protocol in our study were analysed including transitions between activities, resulting in a high time resolution, with the aim to include data of natural behaviours and changes in postures. The analyses of classification accuracy and measurement agreement at the group and individual level provided more insight into the magnitude and source of potential measurement errors, relative to previous analyses in school-aged children. Findings in this study, however, need to be confirmed in free-living conditions as our activity protocol was laboratory-based and might not completely reflect children’s real-world movement patterns and postures. Furthermore, postural allocation by the criterion measure DO might involve some subjectivity, which could have contributed to differences between studies. Another consideration is whether or not our analyses, stratified by age group, were sufficiently powered to detect statistical equivalence. Post-hoc power calculations indicated that a sample size of *n* = 21, *n* = 87 and *n* = 20 for sitting, standing and stepping, respectively, in 5–8y and *n* = 33, *n* = 96 and *n* = 24, respectively, in 9–12y was required. In equivalence testing, if CI’s clearly demonstrate the methods are not equivalent to the reference method, then the sample size is adequate to conclude they are not equivalent. If results are ambivalent (CI’s partial crossing of the equivalence region) and the sample size is not adequate, the results may be at risk of type 2 error. Therefore, the analyses were slightly under-powered to conclude that AP3 estimates of sitting time in 5–8y and standing time in 9–12y were equivalent to DO.

## 5 Conclusion

AP3 demonstrated acceptable accuracy for classifying sitting/lying, standing and stepping in children. Estimates of stepping time were accurate for 5–8y and 9–12y, whereas estimates of sitting/lying time were more accurate in older children. However, AP3 overestimated time spent standing and the absolute number of SB breaks. The group-level accuracy suggests that surveillance applications of AP3 would be acceptable, however, individual level applications might be less accurate.

## 6 Practical implications

• AP3 demonstrated acceptable accuracy for classifying sitting/lying and stepping in school-aged children, but was generally more accurate in 9–12y compared to 5–8y.
• AP3 accurately estimated sitting/lying time in 9–12y and stepping time in 5–8y and 9–12y, however, standing time and the absolute number of SB breaks were overestimated.
• The application of AP3 in school-aged children seems acceptable at the group level, although outcomes of AP3 should be interpreted with caution at the individual level.

## Figures and Tables

**Table 1:** Confusion matrix for classification accuracy (sensitivity) of activPAL3™ (AP3) for postures.

DO	AP3		
	Sitting/lying	Standing	Stepping
Sitting/lying			
5–8y	**0.868**	0.118	0.014
9–12y	**0.953**	0.036	0.011

Standing			
5–8y	0.027	**0.825**	0.148
9–12y	0.019	**0.813**	0.168

Stepping			
5–8y	0.017	0.130	**0.853**
9–12y	0.023	0.131	**0.846**

DO, direct observation.

Values in boldface indicate the proportion of postures correctly classified.
