# Supplementary material for: Validation of thigh-based accelerometer estimates of postural allocation in 5–12 year-olds
Source: J Sci Med Sport. 2017 Mar;20(3):273–7. doi: 10.1016/j.jsams.2016.08.008 (PMC5361055; doi:10.1016/j.jsams.2016.08.008)
Supplement: Supplementary file 1 [file mmc1.docx]

Supplementary Table 1. Activity Protocol

| **Activity Type** | **Activity Trial** | **Intensity** | **Description of Activity Trial** |
| --- | --- | --- | --- |
| Resting | Lying down | Sedentary | Lying down awake on a mattress in supine position - arms at sides - rest for 10 min. |
| Sitting | TV viewing | Sedentary | Watching a movie in a comfortable chair. Instructed to minimise body movements. |
|  | Handheld e-game | Sedentary | Sitting on a chair at a desk playing an e-game on a handheld device. |
|  | Writing/colouring | Sedentary | Sitting on a chair at a desk, 5-8 y: colouring on paper using pencils, 9-12 y: copying words on a pad of paper using a pencil. |
|  | Computer game | Sedentary | Sitting on a chair at a desk playing an educational computer game. |
| Lifestyle | Getting ready for school | Light | Get dressed, set table, pour food, pack up, brush teeth, pack bag, leave for school. |
|  | Standing class activity | Light | Standing activities with minimal movement such as writing/drawing on a white board. |
|  | Dancing | Light | Following a video with dance step instructions (Zumba^®^ fitness). |
|  | Tidy up | Moderate | Tidying up a 4x5 m area: pick up clothes, towels, toys and sport equipment and return them into boxes. |
|  | Basketball | Moderate | Shooting a basketball using a 2.29 m adjustable hoop, chase the ball within a 4.9x4.6 m area and bounce back to the start position at the boundary line apposite from the hoop. |
|  | Soccer | Vigorous | Kicking a foam soccer ball on a 5 m distance between a 1 m wide goal after dodging between a straight line of 5 cones (1 m apart). Instructed to jog back to start position after kicking the ball. |
|  | Locomotor course | Vigorous | Continuously completing a course including 4x 2-foot jump, jogging and sliding between cones around a 4x9.5 m area. |
| Ambulatory | Slow walk | Light | Walking slowly at a self-selected comfortable speed around a 45 m indoor track. Examiner regulates constant speed by recording lap times. |
|  | Brisk walk | Moderate | Walking briskly at a self-selected brisk comfortable speed around a 45 m indoor track. Examiner regulates constant speed by recording lap times. |
|  | Running | Vigorous | Run at a self-selected comfortable speed around a 45 m indoor track. Examiner regulates constant pace by speed lap times. |

All activities are completed for 5 min except for lying down (10 min)

Supplementary Table 2. Participants' characteristics

|  | **5-8y**  **(n=25)** | **9-12y**  **(n=32)** | **Total**  **(n=57)** |
| --- | --- | --- | --- |
| **Age (y)** | 7.0 ± 1.2 | 10.9 ± 1.2 | 9.2 ± 2.3 |
| **Sex** |  |  |  |
| Boys (n) | 11 (44.0%) | 17 (53.1%) | 28 (49.1%) |
| Girls (n) | 14 (56.0%) | 15 (46.9%) | 29 (50.9%) |
| **Height (cm)** | 123.0 ± 8.9 | 146.0 ± 9.2 | 135.9 ± 14.6 |
| **Body mass (kg)** | 24.1 ± 4.0 | 39.4 ± 9.9 | 32.7 ± 10.9 |
| **BMI percentile** | 52.8 ± 24.3 | 53.5 ± 31.9 | 53.2 ± 28.6 |
| Overweight (n) | 2 (8.0%) | 5 (15.6%) | 7 (12.3%) |
| Obese (n) | - | 2 (6.6%) | 2 (3.5%) |
| **Race** |  |  |  |
| Caucasian (n) | 24 (96.0%) | 30 (93.8%) | 54 (94.7%) |
| Asian (n) | 1 (4.0%) | 2 (6.2%) | 3 (5.3%) |

Characteristics of the participants are presented as mean ± SD, distributions of the sample are presented in numbers (n) and percentages.
